# Neuroimaging resilience to stress: a review

**DOI:** 10.3389/fnbeh.2013.00039

**Published:** 2013-05-07

**Authors:** S. J. A. van der Werff, S. M. van den Berg, J. N. Pannekoek, B. M. Elzinga, N. J. A. van der Wee

**Affiliations:** ^1^Department of Psychiatry, Leiden University Medical CenterLeiden, Netherlands; ^2^Leiden Institute for Brain and CognitionLeiden, Netherlands; ^3^Department of Clinical Psychology, Leiden UniversityLeiden, Netherlands

**Keywords:** resilience, stress, neuroimaging, PTSD, MRI, trauma

## Abstract

There is a high degree of intra-individual variation in how individuals respond to stress. This becomes evident when exploring the development of posttraumatic symptoms or stress-related disorders after exposure to trauma. Whether or not an individual develops posttraumatic symptoms after experiencing a traumatic event is partly dependent on a person's resilience. Resilience can be broadly defined as the dynamic process encompassing positive adaptation within the context of significant adversity. Even though research into the neurobiological basis of resilience is still in its early stages, these insights can have important implications for the prevention and treatment of stress-related disorders. Neuroimaging studies contribute to our knowledge of intra-individual variability in resilience and the development of posttraumatic symptoms or other stress-related disorders. This review provides an overview of neuroimaging findings related to resilience. Structural, resting-state, and task-related neuroimaging results associated with resilience are discussed. There are a limited number of studies available and neuroimaging research of resilience is still in its infancy. The available studies point at brain circuitries involved in stress and emotion regulation, with more efficient processing and regulation associated with resilience.

## Introduction

The lifetime prevalence of exposure to severe traumatic events in the United States ranges between 51.2 and 60.7% (Kessler et al., 1995). Individuals who have been exposed to a trauma can develop stress-related psychopathologies, such as depression, anxiety disorders, or posttraumatic stress disorder (PTSD) (Egeland, 1993; Schnyder et al., 2001; Kilpatrick et al., 2003; Bryant et al., 2011). These disorders are a major cause of long-term disability. The United States National Comorbidity Survey found that ~7.8% of the population in the US develops a PTSD at least once in their lives, with women having a higher chance than men (Kessler et al., 1995; Mota et al., 2012). In patients with an anxiety disorder, a higher number of episodes of major depressive disorder (MDD) have been reported in patients with a history of trauma compared to those without the experience of a traumatic event (Zlotnick et al., 1997). Notably, not every individual develops posttraumatic symptoms after experiencing a traumatic event. Many people will experience symptoms of an acute stress disorder, but only a minority will develop PTSD or other affective psychopathologies. Major life-events are also known to be precipitating factors for other psychiatric disorders, and again many individuals will show symptoms of psychological distress, but only a relative minority will develop a disorder like MDD or an anxiety disorder. Whether or not an individual develops posttraumatic symptoms or another stress-related disorder after experiencing a traumatic event can be considered from a summative view, i.e., as being accounted for as a balance between positive and negative influences, affecting most people in the same way or to the same degree (Rutter, 2006), but also from a more dynamic and interactive view. According to this view, both vulnerability and resilience in the context of a specific stressor are higher-order, multidimensional phenomena spanning an individual's biological and psychological profile, developmental history, previous (traumatic) experiences, active choices, social context, current environment, social support, and timing of the traumatic event (Charney, 2004; Feder et al., 2009; Cicchetti, 2010; Holman et al., 2011). Importantly, there is not one universally accepted definition of resilience. Resilience is often more broadly defined as a dynamic process encompassing positive adaptation within the context of significant adversity, and also, from a more psychobiological standpoint, as short- and long-term responses that reduce allostatic load (Charney, 2004; Curtis and Cicchetti, 2007). This definition would differentiate resilience from the concept of resistance. Stress resistance prevents the experience of negative consequences of stressor exposure, whereas stress resilience requires one to experience the negative consequences of stressor exposure in order to demonstrate facilitated recovery from that experience (Fleshner et al., 2011). Moreover, in some individuals the experience of negative effects in response to stressors or adversity may also lead to a decreased vulnerability later in life through a “steeling” or inoculation effect (Rutter, 2012).

Research into psychological factors contributing to resilience is longstanding, and has identified factors such as emotional flexibility, locus of control, social problem solving, and cognitive skills, and several others [for a review see Curtis and Cicchetti (2003)]. More recently, studies have begun to examine biological factors in resilience and their interplay with psychological and environmental factors. In humans, cross-sectional studies have focussed on neuroendocrine and neural markers, while animal models are providing complementing experimental data on behavioral, neural, molecular, and hormonal basis of resilience. Animal data show that in resilient animals there is an absence of the key molecular abnormalities found in susceptible individuals, but also distinct epigenetic and cellular adaptations in response to stressors in various neurotransmitter systems and brain areas (Fleshner et al., 2011; Russo et al., 2012). Insight into biological factors underpinning resilience to stress may open new avenues for prevention and treatment of stress-related disorders (Charney, 2004). In addition, these insights could prove useful in the selection and training of professions known to have a higher risk of trauma exposure (i.e., military personnel, police officers, first responders). Over the past decades neuroimaging has become an increasingly important tool to study neural correlates of adaptive and non-adaptive behavior. Furthermore, neuroimaging allows studying the associations of these neural correlates with other biological factors as well as their interaction with environmental and psychological factors. In addition, neuroimaging facilitates examining the psychobiological mechanisms that underlie these interactions (Meyer-Lindenberg and Tost, 2012).

To investigate neurobiological correlates of resilience to psychological trauma in humans, one would ideally study a group of individuals at baseline, before exposure to trauma, and then assess these individuals repeatedly after exposure to trauma. Those who would have developed sustained symptomatology would then be compared to those who remained symptom-free or only had transient symptoms.

For the sake of this neuroimaging review, we will consider trauma-exposed, non-PTSD (TENP) individuals as resilient subjects. We are aware that defining resilience as the absence of PTSD symptoms after the experience of a traumatic event does not fully cover the multi-dimensional, dynamic nature of the construct of resilience. The term resilience as used throughout our review will therefore be more reflective of the capacity of an individual to avoid negative social, psychological and biological consequences, and cognitive impacts of extreme stress that would otherwise compromise their psychological or physical well-being (Russo et al., 2012). In the present review we will focus on the neuroimaging of resilience to especially severe stressful experiences, such as combat-related trauma, sexual abuse, and sustaining severe injuries through accidents. We will first briefly introduce the most widely used neuroimaging approaches to date. Subsequently, we discuss the brain circuitry of stress and present a review of the available neuroimaging literature on resilience in humans up until 2012.

## Neuroimaging methods

The rapid growth of modern neuroimaging techniques enables us to study both structure and function of the human brain in great detail. Additionally, it allows us to examine the influence of specific biological or specific environmental factors on brain functioning (Meyer-Lindenberg and Tost, 2012). Nowadays, magnetic resonance imaging (MRI) methods are the most widely used tools to examine brain structure and function in living humans, because of the low risks involved, the non-invasive nature of the technique, and the high quality of the obtained images. MRI techniques can be used to localize neuropathological abnormalities or to determine the size or shape of various structures in the brain (Pitman et al., 2001). The MRI-based diffusion tensor imaging (DTI) method can be used to examine white matter tracts. Functional neuroimaging, i.e., dynamic (indirect) measurements of brain activity during rest or during a cognitive, emotional, or pharmacological challenge, can be assessed using functional MRI (fMRI). With fMRI, changes are measured in regional cerebral blood flow based on changes in the concentration of the blood oxygenation level. A relative estimate of the level of activity within a given region of interest can be derived from this blood oxygenation level-dependent (BOLD) signal (Pitman et al., 2001; Huettel et al., 2009). Brain activity and blood flow can also be measured with functional neuroimaging methods that use radioactive ligands such as positron-emission tomography (PET) or single photon-emission computed tomography (SPECT). PET can use radioactive labeled water, oxygen or glucose (Bremner, 2007a; Townsend, 2008). In addition PET and SPECT methods can use radioactive ligands to visualize biochemical elements such as transporters or receptors for certain neurotransmitters.

## Neurocircuitry of stress

Converging data from animal studies, lesion studies in humans and neuroimaging research in healthy controls and patient populations, point at the involvement of specific brain structures and circuitries in the generation of emotional, cognitive, and behavioral responses to stressors and the subsequent regulation of these responses. Key structures involved in this neurocircuitry are the amygdala, insula, hypothalamus, hippocampus, and cortical structures like the medial prefrontal cortex (mPFC) and the anterior cingulate cortex (ACC) (Dedovic et al., 2009).

The amygdala is located in the medial temporal lobes of the brain. It is involved in the encoding and consolidation of emotional memory of events (Bremner, 2007b), and regulates part of the fear response by activating the hypothalamic-pituitary-adrenal axis that releases hormones involved in the stress response. The amygdala can also increase the startle response via connections with the pons in the midbrain and is involved in the modulation of the autonomic nervous system via the hypothalamus (Davis, 1992; Jovanovic and Ressler, 2010). Amygdala activation has been reported in response to positive stimuli as well, suggesting a broad involvement of this structure in emotional arousal (Shin and Liberzon, 2010). Lesions in the amygdala of both humans and rodents have shown that the amygdala is also involved in the elimination of the fear response and emotional behavior (Zola-Morgan et al., 1991; Funayama et al., 2001). The insula is involved in high-level cognitive control and attentional processes (Menon and Uddin, 2010). Additionally, together with the hippocampus, the insula plays a role in processing the context of a potential threat (Gilbertson et al., 2002; Feder et al., 2009). The hippocampus not only has an important role in declarative memory, but is probably also a key-regulator of the hypothalamic-pituitary-adrenal axis activation (Bremner, 2007b). Furthermore, having a small hippocampus could diminish the neuroendocrine regulation, leading to a stronger emotional or hormonal stress response (Gilbertson et al., 2002; Lyons et al., 2007). Animal studies have shown that hippocampal lesions or genetically smaller hippocampi lead to a stronger conditioned fear response and alterations in fear-mediated responses (Phillips and Ledoux, 1992).

The prefrontal cortex (PFC) receives somatosensory, visual and auditory inputs and underlies many cognitive skills. Areas in the PFC have an inhibitory effect on the amygdala (Phelps et al., 2004; Baumann and Turpin, 2010), and the modulation of emotional responsiveness by the PFC through inhibition of the amygdala is supported by lesion studies (Morgan and Ledoux, 1999; Milad and Quirk, 2002). The PFC encompasses many structures, among which the ACC. The ACC is divided into subregions including the dorsal ACC (dACC), the rostral ACC (rACC), and the subgenual ACC (sgACC). Hypofunction of the PFC, the rACC and the sgACC, and hyperactivity of the amygdala and dACC was found to be related to dysregulation of emotion in anxiety or mood disorders (Phan et al., 2005; Shin and Liberzon, 2010). Animal studies have shown that enhanced activation of the infralimbic PFC, the rodent analog of the rACC and sgACC, inhibits the fear response. The ventral-rostral area of the ACC is involved in the processing of emotionally relevant stimuli, while the dorsal-caudal region is more relevant for non-emotional cognitive tasks (Shin et al., 2005, 2007; Mohanty et al., 2007). However, recent studies seem to indicate that both subregions contribute to emotional processing, with ventral-rostral portions of the ACC and the mPFC involved in regulation (Etkin et al., 2011).

**Figure 1:**
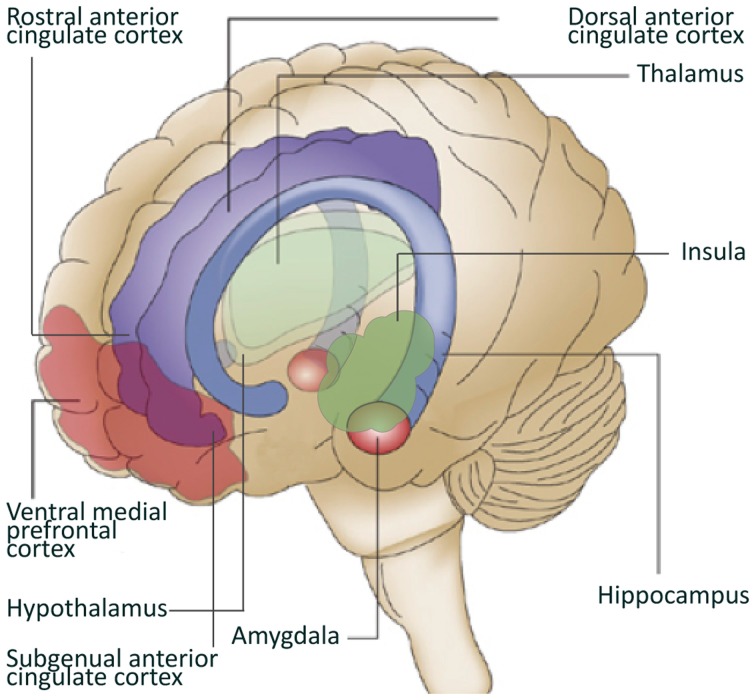
**Brain regions involved in resilience to stress.** Depicted in this figure are brain regions often linked to resilience to stress. Adapted from Schloesser et al. (2008).

With respect to studying stress and resilience to stress, animal studies allow more freedom in manipulating stress responsiveness compared to human research. Interestingly, interventions designed to decrease stress responsiveness by forcing repeated application and selection of the most successful coping strategies have been found to increase the volume of the ventral mPFC (Lyons et al., 2002; Katz et al., 2009), and increase neurogenesis of the hippocampus (Lyons et al., 2010a). In addition, Delgado et al. showed that an inward displacement of the ventral part of the right hippocampus was specific for resilience to stress in rats (Delgado Y Palacios et al., 2011). These findings suggest a firm relationship between successful application of coping strategies and these key structures in the neurocircuitry of stress in animals. For more animal literature on resilience to stress see (Lupien et al., 2009; Lyons et al., 2010b; Franklin et al., 2012) (Figure 1).

## Review

For the current review we conducted a Pubmed search up till 2012, using key terms including: “resilience,” “vulnerability,” “neuroimaging,” “PET,” “SPECT,” “MRI,” “posttraumatic,” “anxiety” “depression” “affective” “stress,” “trauma.” Bibliographies were also reviewed for further citations. We limited our search to studies in humans and in English. Papers were selected based on relevance with a focus on studies in PTSD, but studies on MDD were also included. We also included electronic publications ahead of print.

We will first discuss studies examining structural neural correlates of resilience to stress, followed by studies examining functional neural correlates of resilience to stress, and studies in which the neural correlates of personality factors known to be involved in resilience, i.e., trait-resilience, were taken into account. Table 1 presents the studies that allowed examining resilience, i.e., examined stress-resilient subjects, subjects with psychopathology after stress, and healthy controls without both trauma exposure and PTSD. Because studies in PTSD have informed hypotheses on the neural circuitry involved in resilience, findings from studies in PTSD (comparing PTSD individuals with TENP individuals) will be briefly presented in each section before discussing the results of studies focussing on resilience.

**Table 1:** **An overview of neuroimaging studies specifically examining resilience by using comparisons between three groups: (1) a PTSD group, (2) a TENP group, and (3) a healthy control group without both trauma exposure and PTSD**.

**References**	***n***	**Protocol**	**Findings specific to resilience**
	**PTSD/TENP/HC**		
**THREE-GROUP STUDIES**			
Gurvits et al., 1996	7/7/8	Structural	No resilient specific findings
Liberzon et al., 1999	14/11/14	Trauma-related sounds	No resilient specific findings
Fennema-Notestine et al., 2002	11/11/17	Structural	Smaller frontal and occipital gray matter volumes
Britton et al., 2005	16/15/14	Trauma-script	Decrease in amygdala activation
Falconer et al., 2008	23/17/23	Go/No-Go inhibition task	No resilient specific findings
New et al., 2009	14/14/14	Emotion regulation	Increased activation in medial prefrontal regions during top-down control
Woon and Hedges, 2009	121/77/116	Structural	No resilient specific findings in amygdala volume
Blair et al., 2013	14/15/19	Affective stroop task	Increased activation in medial prefrontal regions during top-down control

**Table:** 

**References**	***n***		**Protocol**	**Findings specific to resilience**
	**PTSD**	**Non-PTSD**		
	**Exposed/non-exposed twins**	**Exposed/non-exposed twins**		
**TWIN STUDIES**				
Gilbertson et al., 2002	17/17	23/23	Structural	Increased hippocampus volume
May et al., 2004	20/23	23/24	Structural	Decreased cavum septum pellucidum size
Kasai et al., 2008	18/18	23/23	Structural	Decreased density in right hippocampus, pregenual ACC, bilateral insulae
Shin et al., 2009	14/14	19/19	Resting-state	Decreased dorsal anterior cingulate activation
Shin et al., 2011	12/12	14/14	Multi-source interference task	Decreased dorsal anterior cingulate activation

HC, Healthy Controls; PTSD, posttraumatic stress disorder; TENP, Trauma-exposed non-PTSD.

## Structural neuroimaging of resilience

We could only identify a very limited number of MRI studies explicitly designed to explore resilience by focussing on its structural correlates. Many studies did not include a healthy, non-exposed control group, which is needed in a cross-sectional design to establish whether differences between exposed groups are related to vulnerability or resilience. Remarkably, there appear to be no studies on resilience using methods to assess white matter integrity or connectivity, such as DTI, or methods to examine structural aspects other than volume or gray matter density, i.e., shape analysis or cortical thickness. Studies usually report on structural aspects of the hippocampus, amygdala, and ACC.

### Hippocampus

One of the core symptoms of PTSD is reliving the traumatic event. With the hippocampus being central in declarative memory, it could be hypothesized that variations in the structure of the hippocampus could contribute to resilience (Bremner, 2007b). Importantly, the hippocampus is also a key-regulator of the hypothalamic-pituitary-adrenal axis activation in response to stressors (Bremner, 2007b). The hippocampus has frequently been studied in PTSD and several studies examining TENP and PTSD subjects found larger volumes of the hippocampus in the TENP subjects (Gurvits et al., 1996; Bremner et al., 2003; Lindauer et al., 2004b; Kitayama et al., 2005; Kasai et al., 2008; Felmingham et al., 2009; Morey et al., 2012), but others did not report this finding (Lindauer et al., 2005; Freeman et al., 2006; Rogers et al., 2009). Smaller hippocampal volumes have also been described in MDD and in healthy “at risk” subjects with a history of depression (Campbell et al., 2004; Videbech and Ravnkilde, 2004).

Because a non-exposed healthy control group was not included in these studies, it is not possible to distinguish whether the differences in hippocampal volume should be attributed to resilience in the TENP group or to the presence of psychopathology or vulnerability in the PTSD group. Controversy exists on the origin of structural differences of the hippocampus in relation to exposure to severe stress. Based on animal studies it has been hypothesized that exposure to traumatic events may damage neurons and inhibit neurogenesis in the hippocampus, showing that the hippocampus is sensitive to the effects of stress (Sapolsky et al., 1990; Bremner, 1999; Golub et al., 2011). This suggests that a smaller hippocampus is a consequence of neurobiological changes associated with extreme or chronic stress. In line with this, smaller left hippocampal volumes were found in women with MDD who have experienced chronic maltreatment in their childhood compared with women with MDD and without childhood maltreatment (Vythilingam et al., 2002). In addition, a decrease in hippocampal volume in MDD has been found to be associated with the duration of depressive episodes (Sheline et al., 1999; Macqueen et al., 2003). In PTSD, psychopharmacological treatment of symptoms has been associated with increases in hippocampal volumes (Vermetten et al., 2003). However, a study in which hippocampal volumes were larger in TENP subjects compared to PTSD subjects, showed that this difference did not change after the PTSD was effectively treated with psychotherapy, suggesting smaller hippocampal volumes to be either a residue or scar, caused by the experience of the trauma, or a factor related to vulnerability, pre-existing the traumatic event (Lindauer et al., 2005). Apart from considerations on how psychotherapy and psychopharmacological treatment influence the brain, an alternative explanation could be that larger hippocampal volume in the TENP is linked to resilience.

To directly address the important controversy, Gilbertson et al. (2002) in an elegant design examined hippocampal volume in a group of PTSD patients and their non-exposed twins, and in combat-related TENP subjects and their non-exposed twins (Gilbertson et al., 2002). It was possible to correct for childhood abuse and alcohol use, factors known to influence hippocampal volume. The authors found smaller hippocampal volumes in both PTSD patients (*n* = 17) and their non-traumatized co-twins (*n* = 17) compared to TENP subjects (*n* = 23) and their non-traumatized co-twins (*n* = 23). In addition, severity of PTSD symptomatology in patients was negatively correlated with the hippocampal volume of both the patients and their trauma-unexposed identical co-twins, suggesting smaller hippocampal volume to be a familial risk factor for developing stress-related psychopathologies. The TENP subjects showed no differences in hippocampal volumes compared to their non-exposed twins. As there were no non-exposed twin pairs in this study, it could not be examined whether the hippocampal volume of the resilient subjects and their co-twins was perhaps larger than average and a potential familial resilience factor. Another, smaller study examined morphometry of the mesial temporal lobe area in adult female victims of intimate partner violence with (*n* = 11) and without (*n* = 11) PTSD, and in non-victimized controls (*n* = 17) (Fennema-Notestine et al., 2002). There were no differences in hippocampal volumes among the three groups. Interestingly, the authors found smaller overall frontal and occipital gray matter volumes in the resilient (violence exposed non-PTSD) subjects, but the interpretation of the findings is difficult because of the small sample and the presence of childhood emotional abuse.

In conclusion, the available data suggest that smaller hippocampal volumes might be the result of exposure to severe stress and perhaps also a vulnerability factor, but it is not clear whether an increased volume is associated with resilience.

### Amygdala

Structural MRI studies in PTSD have also examined structural changes of the amygdala, with some studies reporting a larger amygdala volume in TENP subjects compared to PTSD subjects (Rogers et al., 2009; Morey et al., 2012), while others did not (Gurvits et al., 1996; Bonne et al., 2001; Lindauer et al., 2004b; Kuo et al., 2012).

A recent meta-analytic study, using data from nine different studies in adults, compared the amygdala volumes in PTSD patients (*n* = 121), TENP individuals (*n* = 77), and non-trauma exposed healthy controls (*n* = 116) (Woon and Hedges, 2009). The authors found a larger right amygdala vs. left amygdala in all three groups, which is consistent with a previously conducted study (Pedraza et al., 2004). This suggests that in both PTSD and in resilience to trauma the asymmetry in volume of the amygdala is preserved. In addition, this meta-analysis found no significant differences between amygdala volume in PTSD patients relative to TENP and trauma-unexposed healthy controls (Woon and Hedges, 2009). These results suggest that although the amygdala has a key role in the neurocircuitry of stress, the volume of the amygdala is not associated with influence on vulnerability or resilience toward developing psychopathology after a traumatic event. However, a single case-control study with comparable group sizes to those of the meta-analysis (*n* = 99 PTSD patients; *n* = 101 TENP individuals) found larger left and right amygdalae in the TENP individuals. Evidence of the association between the structure of the amygdala and resilience as well as PTSD symptomatology is currently inconsistent and inconclusive (Morey et al., 2012).

### Anterior cingulate cortex/prefrontal cortex

Studies comparing PTSD subjects with TENP subjects found smaller volumes and lower gray matter density of the ACC in PTSD subjects (Rauch et al., 2003; Woodward et al., 2006; Kasai et al., 2008). More specifically, a smaller volume of the rACC (Rauch et al., 2003; Kasai et al., 2008) and subcallosal cortex was found (Rauch et al., 2003).

Smaller gray matter volumes of the ACC and mPFC have also been found in MDD patients compared to healthy controls, with one of the few longitudinal studies showing loss of volume in MDD in these areas during depressive episodes (Frodl et al., 2008). Similarly, a twin study in subjects with PTSD showed that the gray matter volume reductions were not present in non-PTSD co-twins, suggesting that the reductions are the consequence of the exposure to stress, rather than a possible familial vulnerability factor (Kasai et al., 2008). Furthermore, May et al. (2004) showed in a twin study design a significant reduction of cavum septum pellucidum size in TENP individuals and their co-twins compared to PTSD individuals and their co-twins (May et al., 2004). Increases in the size of the cavum septum pellucidum is linked to impaired limbic development (Raine et al., 2010).

Interestingly, a recent structural neuroimaging study on resilience to MDD examined volumes of the hippocampus, several prefrontal areas, and the basal ganglia in healthy adults without any family history of MDD (*n* = 64), “resilient” healthy individuals with a family history of MDD (*n* = 30), and participants with a current diagnosis of MDD (*n* = 33). A smaller right hippocampal volume, which in PTSD putatively reflects a genetic risk factor, was found in the resilient healthy subjects with a family history of MDD. However, the resilient individuals also showed increased white matter volumes of the right dorsal mPFC as compared to the two other groups. The authors interpreted this as a potential correlate of resilience to stress, possibly linked to the regulatory functions of this region (Amico et al., 2011). To examine the hypothesized modulatory function of emotional responsiveness by the mPFC, Milad et al. (2005) subjected healthy individuals (*n* = 14) to trials of presented pictures of virtual lights followed by an electric shock. In the extinction phase, participants were presented the virtual light without the electric shock. The next day only the virtual lights were presented again and skin conductance was registered as a measure of fear extinction. They found that greater extinction memory (lower skin conductance) was associated with an increased thickness of the ventral mPFC (Milad et al., 2005). These results led the authors to suggest that the size of the ventral mPFC might explain individual differences in the ability to modulate fear. A potential relationship between the ventral mPFC and modulation of emotion responsiveness is further supported by animal studies (Lyons et al., 2002; Katz et al., 2009). These studies used the early handling paradigm [subjecting pups to short periods of separation from their mother during the first week(s) of life] or social separation in order to decrease stress responsiveness and increase successful application of various coping strategies. Animals subjected to this paradigm were found to have an increased volume of ventral mPFC. Other data in rodents show that in animals resilient to certain stress paradigms the expression of certain genes in glutamatergic neurons in the mPFC increases, suggesting increased neuronal activation. This also suggests that the complexity of neuronal architecture in the mPFC increases, which may be mechanisms underlying volume changes (Russo et al., 2012).

## Structural connectivity

Studies examining structural connectivity in PTSD have shown abnormalities of structural integrity of cingulate regions, the cingulum bundle and/or the amygdala, and other frontal regions [for a review see Ayling et al. (2012)]. We did not identify studies in which resilience was or could be explored in humans. In monkeys, the recent study by Katz et al. (2009) not only found increased white matter volumes, but also increased myelination in the mPFC after stress inoculation. This could be the substrate for the decreased stress responsiveness observed in these monkeys, given the role of the ventral mPFC in emotion and arousal regulation (Katz et al., 2009).

Taken into account the limited available structural imaging data from both human and animal studies, the findings most consistently indicate that the (ventral) mPFC volume and structure are associated with resilience, with volumetric and structural alterations reflecting or even underlying increased emotion regulation capacities. For two other key-structures in the stress and emotion circuitry, the hippocampus and the amygdala, the available data suggests associations of structure with vulnerability, but not clearly with resilience.

## Functional neuroimaging

Given the concept of resilience as being a dynamic process, encompassing positive adaptation within the context of significant adversity, studies examining functional correlates of resilience are clearly of importance. One approach is to examine the spontaneous brain activity and its temporal and spatial connectivity in the absence of externally presented tasks or stimuli, so-called resting-state fMRI. Older studies have used PET and SPECT methods; more recently there is an increasing use of resting-state fMRI approaches. With resting-state fMRI several functional networks have been detected, including the default mode network, thought to be involved in autobiographic memory and self-referential processing. With respect to studying resilience, resting-state fMRI can also be applied during anticipation and recovery of stress.

A more widely used functional neuroimaging approach is to study the correlates of brain activity and connectivity while subjects have to engage in a specific emotional or cognitive task, as opposed to the situation in resting-state imaging. Emotional tasks may involve paradigms with specific stimuli associated with a previous stressful or traumatic event, such as trauma scripts to study emotion processing related to the specific event, or with emotional stimuli not related to a previous event to study general emotion processing. In addition, it is also possible to induce psychological and social stress before scanning.

### Resting-state studies

We did not identify PET or SPECT studies in which resilience could be explored, i.e., including a TENP group, a psychopathology and a healthy control group. PET and SPECT studies have found increased amygdala activity at rest in PTSD subjects, with one twin study reporting increased resting metabolic activity as a familial risk factor for PTSD (Chung et al., 2006; Shin et al., 2009).

Only a few resting-state fMRI studies have been performed in PTSD so far, and they seem to point at the importance of resting-state connectivity of different areas and networks involved in self-processing and fear conditioning with an amygdala/ACC circuitry. In a small, but very interesting prospective resting-state fMRI study, Lanius et al. (2010) examined the relationship between connectivity of the default mode network and severity of concurrent and prospective PTSD symptoms in 11 acutely traumatized subjects recruited from emergency departments (Lanius et al., 2010). Participants were assessed at 2, 6, 12, and 36 weeks postaccident and scanning took place at week 6 or 12. A seed-based approach with a seed in the posterior cingulate cortex (PCC)/precuneus region was used. The PCC/precuneus region is implicated in autobiographical memory processes and self-processing operations and a key region in the default mode network (Greicius et al., 2003). Connectivity of this region with the perigenual ACC and the right amygdala was positively correlated with current PTSD symptomatology, whereas the connectivity with the right amygdala predicted symptoms 6 weeks subsequently. The authors interpreted their results as reflecting an increased trauma-related input from amygdala and perigenual ACC circuitry into the default mode network, which could lead to disturbed aspects of self-processing. Less resting-state connectivity between the insula and the right amygdala was shown in a combat-related TENP group compared to PTSD subjects (Rabinak et al., 2011). The insula and amygdala have been shown to be connected during fear conditioning and a reduced resting-state connectivity may underlie less exaggerated fear responses, less persistence of traumatic memories and proneness to affective disorders (Etkin and Wager, 2007).

In another, quite large study comparing TENP (*n* = 72) and PTSD subjects (*n* = 54) recruited from earthquake survivors, the resting-state connectivity of the thalamus was examined. The thalamus is connected to nearly all areas in the cortex and acts as a relay between subcortical areas and the cerebral cortex. The TENP group showed decreased positive connectivity between the thalamus and bilateral inferior and left middle frontal gyri, left inferior parietal lobule, and right precuneus. An increased positive functional connectivity between the thalamus and right medial frontal gyrus and left rACC was also found in this group (Yin et al., 2011).

Of interest for the more dynamic concept of resilience, some resting-state fMRI studies in healthy subjects have aimed to identify patterns of adaptive recovery to laboratory-induced stress. Clearly, this is relevant for elucidating brain mechanisms underlying resilience and vulnerability, as models for stress-related psychopathology usually postulate a loss of the adaptive recovery. Resting-state fMRI seems particularly suited to examine these recovery processes, because brain activity recovery patterns are not disturbed by tasks demands. In a resting-state study in healthy participants, Van Marle et al. investigated poststress amygdala-centered connectivity patterns in order to characterize the aftermath of acute, experimentally induced stress in healthy humans. The investigators recorded resting-state fMRI in 26 female participants immediately following a period of moderate psychological stress induced by means of aversive (vs. emotionally neutral) movie watching. The authors found a prolonged activation in an amygdala-connectivity network after the moderate stress, thought to reflect an extended state of hypervigilance that promotes sustained salience and mnemonic processing after stress (Van Marle et al., 2010).

In another resting-state fMRI study in healthy subjects Veer et al. (2011) examined resting-state functional connectivity during the recovery period after experimentally induced social stress. Forty participants were randomly assigned to the social stress condition or the non-stressful control condition. Resting-state fMRI scans were acquired 60 min after these conditions. In the stressed subjects resting-state fMRI showed an increase in connectivity between the amygdala and the mPFC and between the amygdala and the (PCC)/precuneus region (Veer et al., 2011). The authors interpreted this as showing the top-down inhibitory control by the mPFC and the stress-induced facilitation of self-evaluative processes, involving the default mode network, after or during salient experiences. Both processes can be considered key-elements of the behavioral homeostasis after stress, and this paradigm might be interesting to study adaptive responses in resilient subjects or prospectively.

### Task-related functional studies

As is the case for most other imaging approaches described in this review, task-related functional neuroimaging studies explicitly studying resilience, i.e., using the previously mentioned design with three groups, are scarce. More task-related data are available from studies comparing PTSD subjects with TENP subjects. With disturbed regulation of (emotions evoked by) traumatic memories being a core symptom of PTSD and thought central to its pathophysiology, task-related functional studies typically have employed traumatic memory retrieval scripts to examine alterations in the regulation of emotions induced by traumatic memories. A growing line of research is studying the regulation of non-traumatic induced emotions, focussing on more general emotion regulation capacities in PTSD and TENP. Finally, some recent studies have taken hypervigilance, another core symptom of PTSD in which attention cannot be diverted, as a starting point and studied top-down attentional control systems.

### Studies in PTSD vs. non-PTSD controls

Several functional task-related neuroimaging studies have examined brain activity in PTSD compared to TENP or healthy control subjects [for an extensive review see: Hughes and Shin (2011)]. The most consistent findings in these studies with regard to findings in TENP subjects are an increased activity of the ventral mPFC and rACC, and a relatively lower activity of the amygdala and the dACC as compared to the PTSD subjects during exposure to emotion evocative stimuli. Studies have shown a negative correlation between the increased mPFC and decreased amygdala activity, in line with the regulatory function of mPFC regions over the amygdala. As mentioned above, the first line of research discussed here has used traumatic memory retrieval paradigms, in which subjects are exposed to trauma-related stimuli, such as pictures, sounds, or individual-specific scripts (Liberzon et al., 1999).

Seminal work was done by the group of Bremner et al. (1999a,b) who exposed both Vietnam combat veterans and sexually abused women with and without the diagnosis of PTSD to memories of their trauma during PET scanning and found differences in activity of several brain areas between TENP subjects and PTSD subjects (Bremner et al., 1999a,b). Differences were found in areas involved in emotion regulation, notably in inhibition of the amygdala. In both groups of TENP subjects (war and sexual abuse), an increase in blood flow in the medial prefrontal area, including the subcallosal gyrus, middle temporal gyrus, and right rACC, compared to PTSD patients was found. However, the TENP subjects also showed decreased activity in areas not typically involved in emotion regulation like the PCC, inferior parietal cortex, lingual gyrus, and left precentral gyrus in the motor cortex. In the sexually abused TENP group, there was also increased blood flow in the right hippocampus, inferior fusiform gyrus, supramarginal gyrus, and visual association cortex relative to women with PTSD. This possibly suggests that there may be specific correlates of resilience or vulnerability for specific types of trauma (Bremner et al., 1999a).

More recent studies have predominantly used fMRI paradigms. A study in police officers using traumatic memory retrieval scripts showed an increased activity of the medial frontal gyrus during exposure to trauma scripts in TENP subjects as compared to PTSD subjects (Lindauer et al., 2004a). Traumatic memory retrieval was also used as an fMRI paradigm to examine a group of PTSD and TENP police officers who had experienced the same trauma, but with a (small) subgroup of the PTSD subjects receiving psychotherapy. After therapy symptom scores of this treatment group were similar to those of the TENP group. Subsequent analysis of the scans showed a pattern of increased activity of the mPFC and reduction of amygdala activity during traumatic memory retrieval in the therapy group, comparable to that in the TENP police officers, suggesting a key role for increased emotion regulation capacities in both resilient individuals as well as by therapy (Peres et al., 2011).

Another script-driven fMRI study showed an association between less re-experiencing and less dissociation on the one hand, and activation of the inferior frontal gyrus on the other hand (Hopper et al., 2007). This area was found to be significantly more activated in TENP subjects. Other traumatic script-driven fMRI studies have shown a greater activity of the thalamus region in TENP compared to PTSD patients (Lanius et al., 2001, 2005), which might be interpreted as more efficient information processing capacities in TENP subjects.

Another line of neuroimaging studies in PTSD and TENP subjects examined whether alterations of more general emotion processing capacities are present in PTSD and used paradigms with stimuli unrelated to the specific trauma. Tasks aimed on more general emotion processing included among others pictures of emotional faces (Rauch et al., 2000; Shin et al., 2005), memory tasks with pictures or words unrelated to trauma (Shin et al., 2004; Phan et al., 2006), the emotional counting Stroop task (Shin et al., 2001), or a multi-source interference task (Shin et al., 2011). These studies on more general emotion processing did indeed reveal a similar brain activity pattern in TENP subjects as the studies using trauma-related stimuli, with increased activity of the rACC and ventral mPFC, decreased activity of the amygdala, increased activity of the hippocampus, and a decrease in PCC activity as compared to PTSD subjects. This suggests that more general emotional information processing capacities are involved in vulnerability or resilience.

### Functional studies involving a three-group design with PTSD subjects, TENP subjects, and healthy non-exposed controls

Studies comparing PTSD subjects with TENP point at a central role of emotion regulation brain capacities in the adaptive response to trauma, but the lack of a non-exposed healthy control group does not allow any firm conclusion about whether alterations are specific for resilience or vulnerability. A small number of functional imaging studies have employed task-paradigms and included PTSD, TENP, and a non-exposed healthy control group. Britton et al. performed PET scanning during script driven imagery of emotionally evocative and neutral events in combat-related TENP subjects (*n* = 15), combat-related PTSD subjects (*n* = 16), and healthy controls (*n* = 14) (Britton et al., 2005). Emotionally evocative events included general highly stressful events as well as specific traumatic events. PTSD subjects did not show changes in amygdala activity over conditions, but showed deactivation of the rACC during stressful scripts. Healthy controls showed activation of the amygdala and deactivation of the ventral mPFC during the stressful condition, while the resilient subjects showed the same deactivation of the ventral mPFC. Importantly, they also showed a specific pattern of deactivation of the amygdala during imagery of emotionally evocative events. This can be interpreted as a resilience specific mechanism. However, another study using a similar design with three groups found no patterns of amygdala activity that were specific for the TENP group. Liberzon et al. (1999) used [99mTc]HMPAO SPECT to examine combat-exposed PTSD subjects (*n* = 14), combat-related TENP subjects (*n* = 11), and a group of healthy controls (*n* = 14) and exposed them to white noise and combat noises (Liberzon et al., 1999). Both the TENP group and healthy controls showed less amygdala activation than the PTSD subjects.

Most recent studies with a three-group design have used fMRI paradigms. As the ability to exercise voluntary control over emotional responses was found to be linked to better functioning and emotion regulation in healthy volunteers, and as discussed above, patients with PTSD show less activity in the emotion regulation circuitry when confronted with challenging negative stimuli, several researchers have hypothesized that the capacity to voluntarily or automatically regulate emotions may be a resilience factor (Charney, 2004; Yehuda et al., 2006; New et al., 2009). To directly examine this hypothesis, New et al. (2009) investigated deliberate regulation of emotion in PTSD, TENP, and healthy control groups of 14 women exposed to sexual assault (New et al., 2009). Emotionally neutral and negative pictures were presented, with the negative pictures being not related specifically to sexual assault. The participants had to focus specifically on the deliberate modification (up and down regulating) of emotional responses to the stimuli. Contrary to the general regulation hypothesis, both TENP subjects and PTSD subjects were less capable of downregulation responses to negative stimuli and showed less activity in the lateral PFC compared to healthy controls. However, the TENP subjects were more successful in upregulating their responses to negative stimuli, which was associated with increased activity in the dACC compared to both the PTSD group and the control group. Interestingly, the personality trait “optimism” was significantly correlated with the intensity of ACC activation during voluntary upregulation in TENP subjects compared to both PTSD subjects and healthy controls. This interesting preliminary result suggests that specifically the ability to deliberately engage cognitive-emotional strategies to extinguish negative emotional responses and the functional brain correlates are associated with resilience (New et al., 2009).

A last group of studies has focussed on another core symptom of PTSD, hypervigilance, i.e., the increased attentional bias to environmental threat associated cues and the decreased possibility to focus on other stimuli. Previous work in healthy controls showed that emotional attention involves amygdala priming of representations in the temporal cortex, while the involvement of top-down attentional control systems is needed to divert attention toward task-relevant stimuli and weaken (emotional) responding to (emotional) distracters (Vythilingam et al., 2007).

The few neuroimaging studies that have examined top-down attentional control in PTSD vs. TENP subject or controls did find some alterations, but also for this domain it remains unclear whether it concerns a general deficit or a specific deficit within the context of emotional distracters.

Some studies with a three-group design have tried to address this issue. Falconer et al. (2008) used a Go/No-Go fMRI task to measure inhibitory control of non-emotional stimuli in 23 PTSD patients, 17 TENP individuals, and 23 healthy controls. PTSD subjects showed deficiencies in the recruitment of right inferior frontal cortex and the ventral PFC during inhibitory control. However, there were no activation patterns specific to the resilient TENP individuals (Falconer et al., 2008). A recent twin fMRI study by Shin et al. examined whether functional task-related abnormalities of the ACC, after exposure to severe stress, are acquired characteristics or represent a familial risk. They studied combat-exposed PTSD subjects, their non-exposed co-twins (12 pairs) and combat-related TENP subjects and their co-twins (14 pairs). Subjects performed a cognitive attentional task, the multi-source interference task. Vietnam combat veterans in the TENP group and their identical co-twins showed less task-related dACC activity as compared to Vietnam combat veterans with PTSD and their identical co-twins during the interference task. The dACC activity in the non-exposed twins predicted the severity of the symptomatology in the PTSD subjects (Shin et al., 2011). The results suggest that hyperresponsivity of dACC is a familial risk factor for PTSD. The relative hyporesponsivity in the TENP subjects and their co-twins could be a familial resilience factor.

Recently, Blair et al. (2013) performed an fMRI study on attentional control in 15 TENP individuals, 14 patients with PTSD, and 19 healthy controls. They also explicitly specified a hypothesis on the pattern in the resilient group, expecting resilient subjects to show superior recruitment of regions involved in top-down emotional attention relative to the other two groups during the task performance. Subjects performed the affective number Stroop task, with positive, negative, and emotional pictures selected from the international affective picture system presented as emotional distractors. The PTSD group showed deficiencies in the recruitment of lateral regions of superior and inferior frontal cortex, corresponding with the findings of Falconer et al. (2008), but also a deficiency of recruitment of the parietal cortex that appeared only in the presence of negative distracters (Blair et al., 2013). As hypothesized, the resilient subjects showed an enhanced ability to recruit regions involved in top-down attentional emotional control when compared to the matched healthy controls and the PTSD subjects. Taken together, these studies suggest that deficiencies in the recruitment of especially inferior frontal regions during top-down attentional control in general are specific to PTSD, but resilience specific activity patterns are only present during top-down control of emotional attention.

## Trait resilience

Research has shown that specific personality characteristics contribute to vulnerability and resilience to stress. The Big Five model of personality traits is a widely used model in which individual differences in personality are described in five overall personality factors: neuroticism (also referred to as absence of emotional stability), extraversion, openness, agreeableness, and conscientiousness (Friborg et al., 2005). Studies have shown that neuroticism is a risk factor for the development of PTSD (Breslau et al., 1991; Nakaya et al., 2006). A resilient personality profile was found to consist of low neuroticism, high extraversion, and conscientiousness, but also high scores on openness and agreeableness (Friborg et al., 2005; Campbell-Sills et al., 2006). High trait resilience also coincides with a construct with high scores on optimism, low neuroticism, and behavioral activation sensitivity (Block and Kremen, 1996).

Sub-facets of the Big Five personality traits that are thought to contribute to resilience are high self-esteem, internal locus of control, flexibility in thinking, sense of meaning, and problem-solving skills (Bryant et al., 2011; Daniels et al., 2012). High levels of these traits, together with a fast physiological recovery have been shown to enhance recovery from experimentally induced stress (Tugade and Fredrickson, 2004). Posttraumatic adjustment could also be enhanced by sub-facets of the personality characteristics that include hardiness, believing in having an influence on one's surroundings and also the ability to learn from both positive and negative experiences (Daniels et al., 2012). Furthermore, individuals with high scores on conscientiousness, extraversion and agreeableness are more likely to have a secure and stable environment with supportive social relationships, which also contributes to successful adaptation to stress (Friborg et al., 2005; Daniels et al., 2012).

We identified one study that has examined neural correlates of resilient personality traits, focussing on arousal regulating capacities in healthy volunteers. Waugh et al. (2008) studied the functional neural correlates of trait resilience during anticipation, but also during recovery from threat (Waugh et al., 2008). They operationalized emotional resilience as the flexible and appropriate use of emotional resources. In their event-related fMRI design, healthy participants viewed “threat” cues signaling the possibility of either viewing an aversive picture or a neutral picture, and “non-threat” cues, signaling the viewing of only a neutral picture. High-trait resilient participants exhibited less early and less prolonged insula activity to the neutral pictures shown after a “threat” cue than low-trait resilient participants, indicating quicker and more appropriate adaptation to the neutral stimulus by the high resilient subjects.

In a very interesting small study Daniels et al. (2012) prospectively investigated the neural correlates mediating the relationship between trait resilience and the recovery from a traumatic event. They used a convenience sub-sample of 12 acutely traumatized subjects, derived from a larger sample of 70 acutely traumatized subjects recruited at an emergency department, and fulfilling the DSM-IV PTSD criterion A. Subjects were followed-up for several months to monitor the development of PTSD symptomatology. Trait resilience was assessed with the Connor-Davidson resilience scale (CD-RISC) (Connor and Davidson, 2003). Trait resilience was found to predict a better outcome throughout the first 3 months of follow-up. A trauma script-driven symptom provocation fMRI paradigm with neutral and trauma scripts was used to investigate neural correlates of trait resilience two to four months posttrauma. For imagery of the traumatic vs. the neutral event, CD-RISC scores showed a positive correlation with activity in the right inferior and middle frontal gyrus and the right thalamus. As these regions are known to be involved in arousal regulation and emotional reappraisal, the findings can be interpreted as pointing toward the broader concept of emotion regulation as the mediator between trait resilience and posttraumatic adjustment (Daniels et al., 2012).

## Discussion

Whereas research into psychological factors contributing to resilience is longstanding, only more recently studies have begun to examine biological factors in resilience in humans and their interplay with psychological and environmental factors. Insight into biological factors underpinning resilience to stress may open new avenues for prevention and treatment of stress-related disorders (Charney, 2004). Neuroimaging has become an increasingly important tool to study neural correlates of behavior and to elucidate the role of neural mechanisms in the interaction between genes and environment (Meyer-Lindenberg and Tost, 2012). In a seminal review paper in 2004, Charney stated that with the recent advances it would be possible to create more comprehensive psychobiological models of the ordinary magic of resilience (Charney, 2004). Based on our present review we have to conclude that neuroimaging of resilience is still in its early stages, with only a limited number of studies allowing to specifically examine functional or structural brain characteristics that may contribute to resilience.

Based on findings in studies comparing stress-related psychopathologies, especially PTSD, with healthy controls, the neural circuitry of resilience is usually postulated to overlap with the brain circuitry involved in emotion and stress regulation. Data from imaging studies comparing TENP subjects with PTSD subjects do indeed find important differences in structural and functional characteristics of emotion regulating brain circuitries, putatively underlying or reflecting increased emotion regulation capacities in TENP subjects. By and large, this pattern is also found in the few studies in which subjects with PTSD, TENP subjects and healthy, non-exposed control subjects are compared. Structural studies point at increased gray matter volumes in structures such as the hippocampus, the ventral mPFC, and the rACC and sgACC. Subsequently, functional studies show increased activity in these structures during tasks using emotion evocative stimuli, such as the traumatic script-driven paradigm. The ventral mPFC, rACC, and sgACC exert top-down control over the amygdala and the stress system, which is putatively more efficient or increased in resilience. In addition, the hippocampus is known to be involved in the processing of traumatic experiences, but also in regulation of the stress system. The mPFC also processes traumatic memories and is involved in the regulation of extinction learning and the modulation of fear responses.

Various subregions of the ACC have been found to be involved in emotion regulation, among others through inhibition of the amygdala. In line with this a decreased reactivity of the amygdala together with an increased rACC activity was found in several studies in TENP subjects. The association between resilience and increased regulation of amygdala activation is further supported by a study showing that symptoms of PTSD were very low in combat veterans with unilateral damage in the ventral mPFC or amygdala (Koenigs et al., 2008). It is also thought that adaptive processes after trauma exposure may occur through functional interactions between the mPFC, ACC, and amygdala (Osuch et al., 2008). In line with the findings in humans, a non-human primate study demonstrated alterations of volume and myelination of the ventral mPFC in animal showing reduced stress responsiveness after an inoculation paradigm (Katz et al., 2009). It should be noted that although the majority of the available studies seems to point at the neurocircuitry involved in aspects of emotion and arousal regulation, studies examining functional connectivity do suggest that in resilience, the connectivity of an amygdala-prefrontal network with several other functional networks, such as the default mode network or the salience network, also plays a role.

Another area of neuroimaging research from which insight into possible neural mechanisms underlying resilience may be derived, is that of the neural correlates of personality traits known to facilitate the adaptation to severely stressful situations. This is particularly the case for trait-resilience, a meta-construct involving traits like low neuroticism, high extraversion, and conscientiousness, with several of these traits known to be linked to the characteristics of the neurocircuitry involved in emotion and stress regulation. The few existing neuroimaging studies examining high vs. low-trait resilient subjects found that high trait resilient subjects are characterized by a brain pattern reflecting more efficient arousal modulation and emotional reappraisal. These patterns overlap with the areas and circuitries that were identified in TENP subjects, again suggesting that the broader concept of emotional control and its neural substrate may indeed be pivotal in resilience.

As resilience is probably best conceptualized as a dynamic, context-dependent phenomenon it could be hypothesized that some of the elements specific for resilience only develop during or after the experience of a traumatic event. However, as most available research in humans is cross-sectional, no causal conclusions can be drawn on the temporal order of trauma exposure and brain changes observed in resilient individuals. Moreover, as in the studies conducted so far resilience has been most frequently defined as the absence of PTSD, although data on symptomatology of other trauma-related disorders were gathered in the majority of the discussed studies, the strictness of the exclusion criteria varied between studies. Therefore, it cannot be excluded that the TENP individuals did suffer, to some extent, from (subclinical) depressive or anxiety symptomatology or substance abuse. Hence, it remains uncertain whether the presented findings are specific for the absence of PTSD psychopathology, or for the absence of psychopathology after exposure to trauma altogether.

Given the current state of the art of neuroimaging of resilience as laid out by this review several avenues to gain further insight into the neural mechanisms involved in resilience can be chosen. Ideally, the neural correlates of resilience should be studied longitudinally. Neurobiological and other variables potentially related to resilience would be measured before and after an individual has been exposed to a severe stressor, after which key variables are assessed over time among individuals who develop trauma-related psychopathology, individuals who do not develop psychopathology, and a control group that has not been exposed to trauma. This would allow the identification of baseline “predictors” of resilience as well as that of potential mediators on different levels, i.e., psychological, (epi)genetic, biochemical and neural, and examine their interaction. A homogeneous cohort of subjects for such a longitudinal study is probably most easily recruited amongst first responders or military personnel, who usually are already assessed extensively before active duty. A caveat is that these populations may consist of a selection of resilient individuals. Such a design would also allow to further examine patterns of resilience based on trajectories of psychological complaints after exposure. In addition, this design would enable examination of the temporal stability of neural correlates of resilience (as assessed at baseline) and their malleability by exposure to a severe stressor.

A first approach could be to focus on the structural neural correlates of resilience. Based on our present review of the structural neuroimaging research of resilience, we would postulate resilience to be associated with alterations of gray matter volume and structure of especially the (ventral) mPFC before the exposure to severe trauma. In addition to studying gray matter volume with both region of interest (i.e., mPFC) and whole brain approaches, we suggest to subsequently examine both shape and corticol thickness of the brain. Examining structural connectivity by means of DTI scans would also be an important approach for the structural neuroimaging of resilience. Taking into account the neurocircuitry of stress, the findings in PTSD as well as those in animal studies, the structural connectivity of white matter tracts that connect limbic structures with the ventral PFC and subregions of the ACC would be a clear focus. We would hypothesize increased structural connectivity between these areas, underlying increased emotion regulation capacities, in resilient individuals.

A second approach would be to focus on the functional neural correlates of resilience and their temporal characteristics. Based on our review we believe more general emotion and arousal regulation capacities to underlie resilience, with resilient subjects being especially more capable in upregulating their emotions and having top-down control over emotional attention. However, so far only explicit emotion regulation was examined, and the role of automatic emotion regulation capacities, which may have a stronger neural correlate, has not yet been assessed with functional neuroimaging. In addition to emotion regulation paradigms, we think that a broader design should incorporate novel neuroimaging task paradigms that have not been used to examine functional neural correlates of resilience yet. One would be to visualize the neural activity during acute, non-trauma-related stress, using a paradigm such as the Montreal imaging stress task (Dedovic et al., 2005). We would postulate that during this acute social stress paradigm, resilient individuals exert more control over their limbic system by increasing activity of especially their ventral PFC and rACC. In addition to studying neural characteristics of resilience during stress, neural characteristics of resilience during anticipation, and recovery of stress should be incorporated in the same design. This will allow us to get a complete oversight of neural activity patterns used by resilient individuals in order to process stress over time, from anticipation pre-stress up until recovery during the aftermath of stress. Waugh et al. (2008) already showed patterns of brain activity specific for trait resilient individuals during anticipation and recovery of threat (Waugh et al., 2008). Subsequently, some recent resting-state studies have, as we discussed in the present review, focussed on the immediate adaptive recovery after acute (social) stress. This adaptive recovery, which may be a key element in a more dynamic concept of resilience, was not only associated with involvement of emotion regulation circuitry, but also with that of circuits involved in self-referential processing (Lanius et al., 2010; Veer et al., 2011). Furthermore, based on research from other domains, it can be hypothesized that other brain circuits (e.g., underlying social affiliation, reward dependence, but also higher order cognitive skills) are also involved in resilience and should be investigated in concert (Charney, 2004).

Two other avenues to elucidate neural mechanisms in resilience can be considered as stand-alone cross-sectional designs, but also as baseline assessment in a larger scale, longitudinal pre-post exposure design as described above. One would be to further examine neural correlates of trait resilience, a concept probably directly linked to resilience to psychotrauma. This would theoretically require no special study populations, but so far only a few studies have investigated the structural and functional neural correlates of trait resilience. Another avenue would be to examine the functional and structural effects of stress inoculation or “steeling” paradigms in humans, building on the work in non-human primates. This could not only shed more light on changes in neural mechanisms underlying increased resilience, but may also identify potential neural predictors of response to inoculation. An interesting question would be whether inoculation paradigms in adults result in (inoculation) specific or more general adaptations of especially functional neural mechanisms. We are not aware of any neuroimaging studies that have focussed on the effects of inoculation in humans. Finally, we believe that studies examining the functional and structural correlates of resilience could be enriched by including other neurobiological measures, such as measures for autonomic nervous system reactivity, and by examining the genetic influences on brain structure and function.

In conclusion, several years after the seminal review of Charney (2004), neuroimaging of resilience still seems to be in its infancy, but is expected to benefit from the increasing interest in the “positive” concept of resilience and may be informed by neuroimaging approaches already more widely used in affective disorders and normal behavior, and state-of-the art strategies such as neuroimaging approaches for complex gene-environment interactions (Charney, 2004; Meyer-Lindenberg and Tost, 2012).

### Conflict of interest statement

The authors declare that the research was conducted in the absence of any commercial or financial relationships that could be construed as a potential conflict of interest.
